# An Oldie but Goodie: Lithium in the Treatment of Bipolar Disorder through Neuroprotective and Neurotrophic Mechanisms

**DOI:** 10.3390/ijms18122679

**Published:** 2017-12-11

**Authors:** Eunsoo Won, Yong-Ku Kim

**Affiliations:** Department of Psychiatry, College of Medicine, Korea University, Seoul 02841, Korea; eunsooowon@gmail.com

**Keywords:** lithium, bipolar disorder, therapeutic mechanism

## Abstract

Lithium has been used for the treatment of bipolar disorder (BD) for the last sixty or more years, and recent studies with more reliable designs and updated guidelines have recommended lithium to be the treatment of choice for acute manic, mixed and depressive episodes of BD, along with long-term prophylaxis. Lithium’s specific mechanism of action in mood regulation is progressively being clarified, such as the direct inhibition on glycogen synthase kinase 3β, and its various effects on neurotrophic factors, neurotransmitters, oxidative metabolism, apoptosis, second messenger systems, and biological systems are also being revealed. Furthermore, lithium has been proposed to exert its treatment effects through mechanisms associated with neuronal plasticity. In this review, we have overviewed the clinical aspects of lithium use for BD, and have focused on the neuroprotective and neurotrophic effects of lithium.

## 1. Introduction

Certainly an oldie, lithium was first pharmacologically used in the nineteenth century, known to have a prophylactic effect on recurrent depression [1]. After the anti-manic effects of lithium were discovered, lithium has been used for the treatment of bipolar disorder (BD), in both the acute and maintenance phases of depression and mania for the last sixty or more years [2]. In the process, prescribing rates of lithium had declined at one point [3] due to the growing doubt on lithium’s evidence-based efficacy as earlier studies on lithium did not meet the more recent research standards [4], along with concerns on the possible fatal toxicity and difficulty of use [5]. Furthermore, the changes in BD diagnostic criteria throughout the years [6], and the appearance of newer agents such as valproate and atypical antipsychotics, may also have influenced the use of lithium [7]. However recent studies with designs considered to be more reliable, such as double-blind randomized controlled trials and meta-analyses, and based on contemporary diagnostic criteria such as Diagnostic and Statistical Manual of Mental Disorders, 4th Edition (DSM-IV) and International Statistical Classification of Diseases and Related Health Problems, 10th Revision (ICD-10), have reported lithium to be once again effective in the treatment of BD [8]. Lithium is now considered the treatment of choice for long-term prophylaxis of new episodes [9], regarded as the only substance that prevents both new depressive and new manic episodes [2]. Also, lithium is the only drug with an established anti-suicidal efficacy in BD [10]. Despite such robust treatment effects of lithium, lithium’s specific mechanism of action in mood regulation is still yet to be clarified. Along with the direct inhibition on glycogen synthase kinase 3β (GSK3β), lithium’s various effects on neurotrophic factors, neurotransmitters, oxidative metabolism, apoptosis, neuronal structures and glia, second messenger systems, and biological systems such as the circadian rhythm and hypothalamic–pituitary–adrenal (HPA) axis, have all been suggested to underlie lithium’s therapeutic effects [11]. Although the pathophysiology of BD has not been completely elucidated, a large body of literature indicates BD to be associated with significant neuroanatomical alterations [12]. Such results of studies implicate, a compromised integrity of frontal–subcortical and prefrontal–limbic brain regions, in the pathophysiology of BD [13]. Further evidence suggests changes in the cellular level, including dysregulation of glial–neuronal interactions, to underlie such neuroanatomical alterations [14]. Therefore, lithium has been proposed to exert its treatment effects through mechanisms associated with neuronal plasticity [15]. In this review, we will overview the clinical aspects of lithium use for BD, and focus on the neuroprotective and neurotrophic mechanisms of action of lithium. Furthermore, we have mainly included literature emphasizing modern research designs, and have concentrated on the efficacy of lithium as monotherapy rather than in combination with other agents.

### Methods

We performed an extensive review of the major publications on the clinical aspects of lithium use, lithium’s treatment effects in BD, lithium’s therapeutic mechanism of action, and neuroimaging studies associated with lithium treatment in BD. A comprehensive literature search of peer-reviewed publications was conducted using PubMed, and relevant articles were identified using the following keywords: “lithium” and “bipolar disorder” and “clinical use’, “lithium” and “bipolar disorder” and “treatment effects’, “lithium” and “therapeutic mechanism’, and “lithium” and “bipolar disorder” and “neuroimaging” and “brain structure’. Relevant findings were then identified and synthesized in combination with earlier and extant literature, and referenced articles were further examined to additionally acquire relevant publications. Language was restricted to English, but no time restriction was applied.

## 2. Guidelines to Initiating and Maintaining Lithium

It is well known that before initiating lithium treatment, a number of essential factors should be considered. First, as lithium is excreted by the kidneys, renal functions must be checked, as decreased kidney functions will lead to excess accumulation of lithium and ultimately toxicity [16]. Renal function test should then be maintained every 6 months during treatment, especially the levels of urea and creatinine [17]. Second, thyroid function tests should be administered as lithium has influence on thyroid stimulating hormone, thyroxine, and triiodothyronine [18]. As a result, the prevalence of both overt and subclinical hypothyroidism has been shown to be increased, with circulating thyroid auto-antibodies frequently being found, and thyrotoxicosis also developing [19]. The prevalence of overt hypothyroidism has been reported to vary from 8% to 19% [20], with the main risk factor being female [21]. Thyroid function tests are recommended to be administered at 6 months, and then annually [17]. Third, serum calcium levels should be checked as lithium is known to cause hyperparathyroidism. The prevalence of hyperparathyroidism is estimated to be 7.5% higher than the general population, and has been reported in patients treated with lithium for 15 or more years [22]. Serum calcium level tests should be conducted at 6 months and then repeated annually [17]. Fourth, lithium treatment is often associated with weight gain, with significant weight gain being observed in patients receiving long term treatment [23]. Also, greater weight gain was observed in patients who were already overweight [24]. Weight, along with waist circumference and body mass index should be checked at 6 months and then annually [17]. Furthermore, electrocardiograms may also be recommended as long-term lithium treatment has been associated with corrected QT (QTc) interval prolongation [25]. Recommended medical examinations for safety monitoring with lithium treatment are listed in Table 1.

In addition to the side effects mentioned above which can occur even when within therapeutic lithium levels, such as reduced urinary concentrating ability, hypothyroidism, hyperparathyroidism, and weight gain [26], typical signs and symptoms of lithium intoxication should also be carefully monitored. Lithium plasma levels greater than 1.2 mmol/L are potentially toxic, and in acute intoxication, plasma levels that exceed 2.0 mmol/L can be fatal [27]. Lithium intoxication is mainly presented as central nervous system, gastrointestinal, renal, and cardiovascular symptoms. Central nervous system symptoms include a state of confusion, cerebellar signs such as tremor, dysarthria, ataxia, and nystagmus, extrapyramidal and neuromuscular signs such as fasciculations, fibrillations, and myoclonia, and polyneuropathy. Gastrointestinal symptoms include nausea, vomiting and diarrhea, and renal symptoms include polyuria, polydipsia and nephrogenic diabetes insipidus. Cardiovascular signs include arrhythmia, low blood pressure and rarely shock. Adult respiratory distress syndrome or thermoregulation disturbances may also occur [19]. Signs and symptoms of lithium intoxication are summarized in Table 2.

Although the half-life of lithium in the brain is approximately 24 h, the half-life in plasma is 8 h [28]. A single daily dose of lithium may also be an acceptable option [29,30], but a majority of therapeutic guidelines recommend divided daily doses of lithium, in order to maintain a steady plasma level [31,32]. Two or more daily doses may be applied initially to achieve a standardized 12-h serum lithium level [33], followed by a full daily dose which will aid to enhance compliance and decrease the chance of increased urinary volume [34]. A therapeutic lithium plasma level for BD has been recommended as 0.5–1.2 mmol/L [31], and when initiating treatment, lithium levels should be checked at a steady state, which is at least five days after taking a certain dose, until two consecutive levels within the therapeutic range are established for the same dosage [17]. Lithium has a relatively narrow therapeutic index, and individual differences should be accounted for when initiating treatment as lithium toxicity may occur if levels surpass 1.2 mmol/L. Therefore, lower plasma therapeutic levels at initiation of treatment, such as 0.6–0.8 mmol/L have also been recommended, which can be increased to 0.8–1.0 mmol/L when recurrence occurs [35]. As maintenance therapy, a therapeutic level of 0.4–0.6 mmol/L [31] and up to 0.6–0.8 mmol/L [32] have been recommended. However, it is suggested that plasma level titration is needed according to polarity and symptomatic profile [36], and different prophylactic levels have been recommended for depression-prone BD patients and mania-prone patients, with the levels being 0.4–0.8 mmol/L and 0.6–1.0 mmol/L respectively [37].

Of note, two forms of acquired nonresponse to lithium that develop over the treatment course in patients who have previously shown adequate response have been reported. The first type of nonresponse is discontinuation-induced refractoriness, is when patients who have showed good long-term response to lithium then discontinue treatment, and after experiencing a major recurrence, do not respond to lithium at previously effective doses [38]. The second type of nonresponse is the development of a pharmacodynamic tolerance to lithium, described as mild, brief, or infrequent symptoms starting to occur then progressively increasing in severity, duration, or frequency, until the original pattern of illness prior to treatment recurs, even while maintaining lithium treatment [39]. Physicians should be aware of the possibility of occurrence of the two phenomena while maintaining treatment in patients with BD, and apply appropriate management of the conditions when necessary.

## 3. Lithium in the Treatment and Prophylaxis of Bipolar Disorder (BD)

Lithium has been reported by recent literature to be effective in treating manic and mixed episodes compared to placebo, including numerous randomized controlled trials (RCTs) [40,41,42] and meta-analyses [43,44,45]. When compared to other mood stabilizers and atypical antipsychotics, lithium was shown to have similar treatment efficacies in acute manic and mixed episodes as valproate [46,47], olanzapine [48], risperidone and haloperidol [49]. On the other hand, manic or mixed patients taking lithium were shown to have less response but no significantly different remission rates compared to patients taking olanzapine [50]. Also, manic but not mixed or rapid cycling patients taking lithium were reported to show less response and a lower remission rate compared to those who were on quetiapine [51]. Lithium was also suggested to be more effective in manic patients compared to valproate [52] and olanzapine [53]. One RCT reported an equal efficacy of verapamil compared with lithium in the treatment of mania [54]. Studies of adequate quality comparing the efficacy of lithium with other anticonvulsants and mood stabilizers such as oxcarbazepine, lurasidone, gabapentin and tiagabine, have not yet been conducted [8]. Furthermore, no RCTs have yet investigated the treatment effects of lithium in purely mixed or hypomanic patients, with a systematic review suggesting lithium monotherapy to lack significant prophylactic benefits in mixed episodes [55]. Concerning onset of action, lithium has been reported to have a slower onset of action compared to antipsychotics such as haloperidol, olanzapine and risperidone, with lithium being 6~10 days [56] and antipsychotics being 2~6 days [57].

Although only a few RCTs have been conducted on the treatment effects of lithium in bipolar depression, several treatment guidelines recommend lithium as a first-line treatment agent for bipolar I disorder (BD-I) depression [58]. Possibly considered a disadvantage, lithium has been shown to have less efficacy in treating bipolar depression compared to quetiapine and antidepressants such as venlafaxine by previous studies. A double-blind, placebo-controlled study of quetiapine and lithium monotherapy in the acute phase of BD-I or bipolar II disorder (BD-II) major depression reported quetiapine, but not lithium, to have significant treatment efficacy compared to placebo [59]. A subsequent open-label, randomized study on the treatment effects of quetiapine or lithium monotherapy in patients with BD-I or II major depression reported both agents to reduce depressive symptoms, but the remission rate to be higher with quetiapine [60]. A randomized, parallel group, open-label trial on venlafaxine and lithium monotherapy in rapid and non-rapid cycling patients with BD-II depression reported venlafaxine to have superior efficacy compared to lithium independent of cycling status, and to not result in a higher proportion of mood conversions [61]. On the other hand, lithium has shown equal efficacy compared to other medications such as lamotrigine, as a single blind study of lithium and lamotrigine treatment for BD-II depression reported both agents to be effective with similar response and remission rates [62]. Furthermore, considered as advantages of lithium therapy, a protective effect against switching to mania when treating bipolar depression [63], and a reduction in the risk of suicidal behavior which is often accompanied in bipolar depression [64], have been suggested with lithium treatment. However, the antidepressant effect of lithium has been reported to have a delayed onset of 6–8 weeks. Therefore, in clinical practice, a combination of lithium with other agents which are considered to be effective in the treatment of bipolar depression, is more likely to be administered, and despite the insufficient evidence, lithium remains important in the treatment of bipolar depression [65].

Lithium has been shown to be effective in the prophylaxis of both mania and bipolar depression [66], hence the majority of treatment guidelines recommend lithium as a first-line agent for maintenance therapy. A RCT including patients with BD-I and a current or recent manic, depressive, or mixed episode reported lithium to significantly increase time to recurrence of both manic events and depressive events compared with placebo [67]. A randomized open-label trial including patients with BD-I and were not having an acute episode but were indicated for long-term therapy, reported lithium monotherapy to be more likely to prevent relapse than valproate monotherapy [66]. Based on previous evidence, lithium has been suggested to provide better prophylactic efficacy against manic episodes compared to depressive episodes [8].

As BD is often presented with psychotic features especially during manic episodes, the efficacy of lithium treatment on bipolar psychosis has previously been investigated [68]. However, only a few studies have systematically evaluated lithium’s treatment effects on mania with psychosis. Studies comparing lithium with first and second-generation neuroleptics in mania with psychosis reported chlorpromazine to be superior than lithium in treating psychotic features [69], and aripiprazole but not lithium, to be more effective than placebo in treating psychotic symptoms [40]. On the other hand, lithium was shown to have similar efficacy as quetiapine, and to be superior to placebo in the treatment of psychosis in manic episodes [41]. Furthermore, lithium monotherapy was shown to be significantly better than placebo in improving mania in a psychotic subtype [70], to produce early improvement of psychotic symptoms, and to have similar efficacy in both psychosis and non-psychosis mania [71]. Future research on lithium’s anti-psychotic properties in the treatment of BD should be conducted.

The anti-suicidal effects of lithium in BD have previously been reported by numerous RCTs and meta-analyses. A meta-analysis published in 2006 which included 31 studies and 33,340 subjects, reported risks of completed and attempted suicide to be lower during lithium treatment in patients with BD and major depression [72]. In a meta-analysis published in 2009, which included 6 studies that directly compared patients with BD who were treated with either lithium or anticonvulsants (carbamazepine, divalproex, lamotrigine), reported suicidal acts to be significantly lower during treatment with lithium [73]. A meta-analysis published in 2013 which included 48 RCTs, also reported lithium to be more effective than placebo in reducing the number of suicides in patients with mood disorders, although no clear benefits were observed for lithium in preventing deliberate self-harm [64]. Not only considered a consequence of lithium’s mood stabilizing effects, the anti-suicidal effects of lithium have also been associated with the reduction of impulsivity and aggressiveness, both of which are associated with an increased risk of suicide [74], and are often present in bipolar depression or mixed states [75]. Furthermore, lithium’s numerous mechanisms of action, including the inhibition of GSK3β, have been suggested to contribute to lithium’s anti-suicidal properties [76]. The therapeutic mechanisms of lithium are discussed in detail in the below section.

## 4. Therapeutic Mechanisms of Lithium

Although the specific therapeutic mechanisms of lithium in mood regulation has not been clarified, lithium is recently being suggested to exert its mood stabilizing effects by acting on cellular targets and exerting neuroprotective effects [77]. The GSK-3 signaling pathway modulates apoptosis and synaptic plasticity, with increased activity supporting apoptosis, and attenuated activity enhancing neuroplasticity and cellular resilience [78]. Manipulation of the GSK-3 pathway has been shown to produce both antimanic and antidepressant effects [79], and genes regulating GSK-3 have been implicated in BD etiology [80,81]. Lithium is considered to influence numerous neuroprotective pathways through increasing phosphorylated GSK3β and inhibiting its action, ameliorating the effects of excitotoxicity [82]. Previous studies have reported treatment response to lithium to be predicted by *GSK3β* gene expression and phosphorylation [83,84], and lithium induced increases in phosphorylated GSK3β to be correlated with symptom improvement [82]. Brain-derived neurotrophic factor (BDNF) is well known for its involvement in neuronal maturation, differentiation and survival, synaptic plasticity, and long-term memory consolidation, and is highly expressed in the cerebral cortex and hippocampus [85]. Numerous studies have reported decreased BDNF levels in patients with bipolar depression and mania, along with low levels of BDNF to be correlated with severity of depression and mania symptoms [86,87]. *BDNF* gene polymorphisms have also been associated with the risk for BD, early onset of the disease, suicidality, propensity toward rapid cycling, and treatment response [88,89]. Lithium has been suggested to prevent cellular degeneration through BDNF upregulation, with chronic lithium treatment shown to increase BDNF [90].

Mitochondrial function is known to be essential for regulating neuroplasticity, apoptosis, and intracellular calcium levels. Changes in endocellular calcium have an essential role in modulating intracellular signaling cascades and neurotransmitter release [91]. Defective mitochondrial function has been associated with abnormal oxidative metabolism and damage to desoxyribonucleic acid (DNA), which contributes to neuronal apoptosis [92,93]. Mitochondrial dysfunction has been implicated in BD, with hippocampal expression of genes related to mitochondrial proteins being reduced in patients with BD [93]. Furthermore, accelerated telomere shortening observed in BD, has been suggested to be greatly influenced by stress-related oxidative damage [94]. Lithium has been shown to have anti-oxidative effects through various mechanisms such as increasing the antioxidants [95], reducing the expression of stress proteins [96], reducing proinflammatory molecules and attenuating immune responses to stress [97,98,99,100], and influencing the expression of genes involved in oxidative cytoprotection [101]. Studies conducted on patients with BD have reported lithium to decrease lipid peroxidation levels [102], and ameliorate mitochondrial dysfunction, which reverses the effects of oxidative stress [103]. Previous studies have reported lithium to prevent apoptosis by modulating GSK3β [104], tumor protein p53 [105] and B-cell lymphoma 2 [106], to increase neurons, glial cells and astrocyte density in brain areas such as the hippocampus [107], stimulate re-myelination and repair demyelinated pathways [108]. Furthermore, various other second messenger systems are thought to be involved with the therapeutic effects of lithium, including the phosphoinositide cycle, protein kinase C, and intracellular Ca^2+^ [109]. Lithium is considered to ameliorate inositol depletion-related mitochondrial dysfunction by inhibiting inositol monophosphatase 1 [110], to enhance reparative neuronal plasticity by inhibiting protein kinase C through a myristoylated alanine-rich C kinase substrate pathway [111], to maintain Ca^2+^ homeostasis by downregulating the transient receptor potential channel 3 [112], and to enhance cyclic adenosine monophosphate (cAMP)-induced cAMP response element-binding protein (CREB) dependent gene transcription [113].

Disturbances in various neurotransmitter systems have been reported in BD. Numerous studies including meta-analyses have suggested glutamatergic dysregulation to underlie the pathophysiology of BD, with elevated levels of glutamate-related metabolites being observed in prefrontal–limbic brain areas [114], and such glutamatergic dysregulation suggested to reflect glial abnormalities [115]. Also, the expression of subunits of NMDA glutamate receptors in the hippocampus, has been shown to be decreased in BD [116]. Dopaminergic dysfunction has also been implicated in BD, with excessive dopaminergic activity in mania precipitating dopamine receptor down-regulation, and inducing a transition to depression [117]. Previous studies have also reported abnormalities in gamma-aminobutyric acid (GABA) transmission in BD, with alterations in GABA platelet uptake [118] and GABA transmission [119], along with increases in GABA/creatinine ratio [120]. Lithium has been shown to modulate such neurotransmitters including glutamate, dopamine, GABA, acetylcholine and glycine [109]. For glutamate, lithium has been reported to act on presynaptic terminals and inhibit excitatory postsynaptic currents [121], increase enhancers and promoters of genes associated with glutamate neurotransmission [122], decrease phosphorylation of *N*-methyl-d-aspartate (NMDA) receptor subunits post-synaptically [123], along with NMDA-induced cytoskeletal deterioration [124]. In patients with BD, lithium has been shown to have a bimodal action in hippocampal glutamate concentration depending on the plasma levels [125]. For dopamine, lithium is considered to exert a regulatory effect and has been shown to prevent excessive dopamine release [126]. For GABA, lithium has been reported to influence its neurotransmission, but the effects being less, compared to glutamate [121]. For acetylcholine and glycine, lithium was recently reported to have influence on both neurotransmitters, by attenuating depressive behaviors through cholinergic pathways [127], and differentiating the expression of glycine transporters on neural cell surfaces [128]. 

There has been an increase in findings on lithium’s effects on circadian rhythms and the HPA axis, which are both considered to be associated with the pathophysiology of BD. A significant amount of evidence indicate an association between circadian dysregulation and BD. Alterations in sleep structure such as increased REM sleep density, sleep pattern variability, sleep latency, sleep duration, number of arousals, and fragmented sleep, along with decreased sleep efficiency have been implicated in BD [129,130]. Alterations in endocrine and neurotransmitter diurnal rhythms have also been suggested in BD, with changes in melatonin secretion [131] and diurnal glucocorticoid regulation being reported. Associations between circadian genes such as *TIMELESS*, *ARNTL1*, *PER3*, *NR1D1*, *CLOCK*, and *GSK3β*, and BD have also been suggested [130]. Lithium has been shown to resynchronize circadian rhythms [132] by modulating clock gene expression [133], and the treatment effects of lithium have been shown to be mediated by circadian components [134]. Alterations in HPA axis function in BD include excess corticotropin-releasing factor (CRF) and adrenocorticotropic hormone (ACTH) secretion, which ultimately increase cortisol levels [135]. Furthermore, decreased sensitivity of glucocorticoid receptors which leads to the disruption of the HPA axis feedback regulation, has also been implicated in BD [136]. Lithium has been suggested to activate the HPA axis, as lithium’s influence on protein kinase C can in turn influence the expression of corticotrophins in the adrenal glands [137].

## 5. Lithium and Brain Structure

The majority of changes in the cellular level, neurotransmitter and neurotrophic systems [138,139] indicate alterations in neuroplasticity to underlie the pathophysiology of BD. In order to elucidate the neuroanatomical phenotype of BD, numerous imaging studies have been conducted, although the results have been frequently equivocal and non-replicable. However, certain brain regions have constantly been implicated in BD. Increased lateral ventricle volume that correlates with number of episodes, has been identified in patients with BD, which indicates BD to be progressively deleterious to the brain [140,141]. The ventromedial prefrontal cortex (PFC) is connected to limbic structures, and is involved in processing emotionally relevant information. Decreases in volume of the ventromedial PFC have been shown in adolescent patients with BD, but equivocal findings have been reported in adult patients [142]. The dorsolateral PFC, which is part of an executive–cognitive network that regulates limbic structures, has been shown to be decreased in thickness in patients with BD [143]. The anterior cingulate cortex (ACC) connects prefrontal cortical areas with subcortical limbic regions, and plays an important role in cognitive–emotional integration. Volume reductions in the ACC have been reported in patients with BD. Inconsistent findings regarding hippocampal volume exist, with increased, decreased and no difference in volume being observed in patients with BD [144]. As amygdala volume has been reported to differ according to age group in BD, with smaller volumes being observed in adolescent patients, and larger volumes being observed in adult patients, it is suggested that structural changes of the amygdala may reflect progression of the disease [145]. Alterations of volume of other subcortical regions, including the nucleus accumbens, putamen and caudate have also been reported, but with mixed results [142]. Alterations in white matter (WM) integrity have also been widely reported by numerous diffusion tensor imaging studies in patients with BD. WM tracts connecting the ACC with the amygdala and hippocampus, and the frontal lobe with the insula, amygdala, hippocampus, occipital lobe, thalamus and cingulate gyrus, have all been observed [146,147]. Such compromised integrity of frontal–subcortical and prefrontal–limbic brain regions, may support an organic basis for the symptomatology of BD.

In order to investigate the putative neurotrophic and neuroprotective effects of lithium, numerous imaging studies have been conducted for the last decade. Although previous studies have also reported no effect of lithium on brain structure [148,149,150], numerous studies have reported positive effects of lithium on gray matter (GM) volume and WM integrity. BD patients who were treated with lithium showed significantly greater GM density compared to healthy controls in diffuse cortical regions, particularly in the cingulate and paralimbic cortices, with the neurotrophic effects of lithium suggested as a possible etiology of such neuroanatomic differences [151]. Lithium was also shown to attenuate the decrease in both GM and WM in BD patients [152]. Daily dosage of lithium treatment was positively correlated with superior temporal gyrus volume in patients with BD [153], and BD patients on lithium also showed increased GM in the subgenual anterior cingulate gyrus, postcentral gyrus, hippocampus/amygdala complex and insula compared to patients on other treatment agents [154]. Significantly decreased thalamus volume in lithium-free BD patients was reported compared to healthy controls, whereas lithium-treated BD patients showed no difference to the healthy control group [155]. In patients with BD depression, long-term lithium treatment was associated with increased GM volumes including the dorsolateral PFC, orbitofrontal cortex, ACC, superior temporal cortex, parieto-occipital cortex, and basal ganglia [15]. BD patients with over two years of lithium treatment showed hippocampal volumes comparable to controls, whereas patients with limited lifetime lithium exposure showed significantly lower hippocampal volumes compared to controls [156], with the findings being replicated in a subsequent study [157]. Imaging genetics studies have been conducted on the influence of lithium and *GSK3β* promoter rs334558 polymorphism on brain structure in patients with BD. The less active *GSK3β* rs334558*C gene-promoter variant and long-term administration of lithium, were associated with increased WM integrity in regions including the corpus callosum, forceps major, cingulum, superior and inferior longitudinal fasciculus, inferior fronto-occipital fasciculus, posterior thalamic radiation, superior and posterior corona radiata, and corticospinal tract [158]. Lithium treatment and the *GSK3β* promoter rs334558 polymorphism was also synergistically associated with increased GM volumes including the subgenual and orbitofrontal cortex in patients with BD depression [159]. A recent meta-analysis on GM volume in BD patients with and without lithium treatment reported global GM volume to be significantly larger in lithium-treated BD patients compared to lithium-free patients [160]. However, due to the lack of correlations between alterations in certain GM and WM structures and improvement of BD symptoms, brain structural changes associated with lithium treatment may not always be interpreted as direct results of the therapeutic effects of lithium, and the findings from imaging studies should be interpreted with caution.

## 6. Conclusions

BD is a heterogeneous condition with a myriad of symptoms varying in manifestation, and the dysregulation of numerous biochemical pathways have been suggested to be involved in the pathogenesis of BD. As the complex therapeutic mechanisms of lithium are gradually becoming unraveled, it is becoming more evident that the cellular mechanisms and biological systems modulated by lithium, are deeply intertwined with the biological disruptions implicated in BD. Therefore, a deeper and clearer understanding of the specific therapeutic mechanisms of lithium, will aid us to establish a better understanding of the complex mechanisms underlying the pathophysiology of BD. Although it is undeniable that lithium is effective in the treatment and prophylaxis of BD, certain subtypes of the disorder seem to respond less to lithium, and not all patients benefit from its treatment effects. The identification of biologic predictors of lithium response remains an important step in the pursuit of personalized medicine for the treatment of BD [161]. As current neuroimaging techniques enable us to study the underlying neurobiological mechanisms of BD, along with the neuroprotective and neurotrophic effects of lithium, future imaging studies are anticipated to facilitate the identification and investigation of biologic predictors of lithium response. The fact that lithium has withstood several ordeals, such as the concerns on its possible fatal toxicity and difficulty of use, and has stayed strong as the treatment of choice for BD for over six decades, proves lithium to be a goodie. Even Nirvana made a song about it.

## Figures and Tables

**Table 1:** Recommended medical examinations for safety monitoring with lithium treatment.

Recommended Medical Examination	Time of Examination
Renal function test	Baseline, every 6 months
Thyroid function test	Baseline, at 6 months, annually
Calcium	Baseline, at 6 months, annually
Weight, waist circumference, body mass index	Baseline, at 6 months, annually
Electrocardiogram recommended for the risk of QTc interval prolongation	

**Table 2:** Signs and symptoms of lithium intoxication.

Symptoms	Lithium Intoxication > 1.2 mmol/L
Central nervous system	State of confusion
	Cerebellar signs (tremor, dysarthria, ataxia, nystagmus)
	Extrapyramidal and neuromuscular signs (fasciculations, fibrillations, myoclonia)
	Polyneuropathy
Gastrointestinal	Nausea, vomiting, diarrhea
Renal	Polyuria, polydipsia, nephrogenic diabetes insipidus.
Cardiovascular	Arrhythmia, low blood pressure, shock.
Adult respiratory distress syndrome, Thermoregulation disturbances	

## References

[1] Shorter E. (2009). The history of lithium therapy. Bipolar Disord..

[2] Licht R.W. (2012). Lithium: Still a major option in the management of bipolar disorder. CNS Neurosci. Ther..

[3] Blanco C., Laje G., Olfson M., Marcus S.C., Pincus H.A. (2002). Trends in the treatment of bipolar disorder by outpatient psychiatrists. Am. J. Psychiatry.

[4] Moncrieff J. (1997). Lithium: Evidence reconsidered. Br. J. Psychiatry.

[5] Young A.H., Hammond J.M. (2007). Lithium in mood disorders: Increasing evidence base, declining use?. Br. J. Psychiatry.

[6] Deshauer D., Fergusson D., Duffy A., Albuquerque J., Grof P. (2005). Re-evaluation of randomized control trials of lithium monotherapy: A cohort effect. Bipolar Disord..

[7] Jefferson J.W. (2005). Old versus new medications: How much should be taught?. Acad. Psychiatry.

[8] Curran G., Ravindran A. (2014). Lithium for bipolar disorder: A review of the recent literature. Expert Rev. Neurother..

[9] Geddes J.R., Miklowitz D.J. (2013). Treatment of bipolar disorder. Lancet.

[10] Baldessarini R.J., Tondo L., Hennen J. (2003). Lithium treatment and suicide risk in major affective disorders: Update and new findings. J. Clin. Psychiatry.

[11] Stahl S.M. (2013). Stahl’s Essential Psychopharmacology: Neuroscientific Basis and Practical Applications.

[12] Kempton M.J., Salvador Z., Munafo M.R., Geddes J.R., Simmons A., Frangou S., Williams S.C. (2011). Structural neuroimaging studies in major depressive disorder. Meta-analysis and comparison with bipolar disorder. Arch. Gen. Psychiatry.

[13] Maletic V., Raison C. (2014). Integrated neurobiology of bipolar disorder. Front. Psychiatry.

[14] Ongur D., Drevets W.C., Price J.L. (1998). Glial reduction in the subgenual prefrontal cortex in mood disorders. Proc. Natl. Acad. Sci. USA.

[15] Benedetti F., Radaelli D., Poletti S., Locatelli C., Falini A., Colombo C., Smeraldi E. (2011). Opposite effects of suicidality and lithium on gray matter volumes in bipolar depression. J. Affect. Disord..

[16] Gitlin M. (1999). Lithium and the kidney: An updated review. Drug Saf..

[17] Ng F., Mammen O.K., Wilting I., Sachs G.S., Ferrier I.N., Cassidy F., Beaulieu S., Yatham L.N., Berk M., International Society for Bipolar Disoeders (2009). The international society for bipolar disorders (ISBD) consensus guidelines for the safety monitoring of bipolar disorder treatments. Bipolar Disord..

[18] Lombardi G., Panza N., Biondi B., Di Lorenzo L., Lupoli G., Muscettola G., Carella C., Bellastella A. (1993). Effects of lithium treatment on hypothalamic-pituitary-thyroid axis: A longitudinal study. J. Endocrinol. Investig..

[19] Grandjean E.M., Aubry J.M. (2009). Lithium: Updated human knowledge using an evidence-based approach: Part III: Clinical safety. CNS Drugs.

[20] Kleiner J., Altshuler L., Hendrick V., Hershman J.M. (1999). Lithium-induced subclinical hypothyroidism: Review of the literature and guidelines for treatment. J. Clin. Psychiatry.

[21] Johnston A.M., Eagles J.M. (1999). Lithium-associated clinical hypothyroidism. Prevalence and risk factors. Br. J. Psychiatry.

[22] Bendz H., Sjodin I., Toss G., Berglund K. (1996). Hyperparathyroidism and long-term lithium therapy—A cross-sectional study and the effect of lithium withdrawal. J. Intern. Med..

[23] Keck P.E., McElroy S.L. (2003). Bipolar disorder, obesity, and pharmacotherapy-associated weight gain. J. Clin. Psychiatry.

[24] Vendsborg P.B., Bech P., Rafaelsen O.J. (1976). Lithium treatment and weight gain. Acta Psychiatr. Scand..

[25] Mamiya K., Sadanaga T., Sekita A., Nabeyama Y., Yao H., Yukawa E. (2005). Lithium concentration correlates with QTc in patients with psychosis. J. Electrocardiol..

[26] McKnight R.F., Adida M., Budge K., Stockton S., Goodwin G.M., Geddes J.R. (2012). Lithium toxicity profile: A systematic review and meta-analysis. Lancet.

[27] Malhi G.S., Tanious M., Gershon S. (2011). The lithiumeter: A measured approach. Bipolar Disord..

[28] Plenge P., Stensgaard A., Jensen H.V., Thomsen C., Mellerup E.T., Henriksen O. (1994). 24-hour lithium concentration in human brain studied by Li-7 magnetic resonance spectroscopy. Biol. Psychiatry.

[29] Jensen H.V., Plenge P., Mellerup E.T., Davidsen K., Toftegaard L., Aggernaes H., Bjorum N. (1995). Lithium prophylaxis of manic-depressive disorder: Daily lithium dosing schedule versus every second day. Acta Psychiatr. Scand..

[30] Malhi G.S., Tanious M. (2011). Optimal frequency of lithium administration in the treatment of bipolar disorder: Clinical and dosing considerations. CNS Drugs.

[31] American Psychiatric Association (2002). Practice guideline for the treatment of patients with bipolar disorder (revision). Am. J. Psychiatry.

[32] Yatham L.N., Kennedy S.H., O’Donovan C., Parikh S., MacQueen G., McIntyre R., Sharma V., Silverstone P., Alda M., Baruch P. (2005). Canadian Network for Mood and Anxiety Treatments (CANMAT) guidelines for the management of patients with bipolar disorder: Consensus and controversies. Bipolar Disord..

[33] Amdisen A. (1973). Serum lithium estimations. Br. Med. J..

[34] Bowen R.C., Grof P., Grof E. (1991). Less frequent lithium administration and lower urine volume. Am. J. Psychiatry.

[35] National Collaborating Centre for Mental Health (UK) (2006). Bipolar Disorder: The Management of Bipolar Disorder in Adults, Children and Adolescents, in Primary and Secondary Care.

[36] Kleindienst N., Severus W.E., Greil W. (2007). Are serum lithium levels related to the polarity of recurrence in bipolar disorders? Evidence from a multicenter trial. Int. Clin. Psychopharmacol..

[37] Severus W.E., Kleindienst N., Evoniuk G., Bowden C., Moller H.J., Bohus M., Frangou S., Greil W., Calabrese J.R. (2009). Is the polarity of relapse/recurrence in bipolar-I disorder patients related to serum lithium levels? Results from an empirical study. J. Affect. Disord..

[38] Fornaro M., Stubbs B., De Berardis D., Iasevoli F., Solmi M., Veronese N., Carano A., Perna G., De Bartolomeis A. (2016). Does the “silver bullet” lose its shine over the time? Assessment of loss of lithium response in a preliminary sample of bipolar disorder outpatients. Clin. Pract. Epidemiol. Ment. Health.

[39] Post R.M. (2012). Acquired lithium resistance revisited: Discontinuation-induced refractoriness versus tolerance. J. Affect. Disord..

[40] Keck P.E., Orsulak P.J., Cutler A.J., Sanchez R., Torbeyns A., Marcus R.N., McQuade R.D., Carson W.H., Group C.N.S. (2009). Aripiprazole monotherapy in the treatment of acute bipolar I mania: A randomized, double-blind, placebo- and lithium-controlled study. J. Affect. Disord..

[41] Bowden C.L., Grunze H., Mullen J., Brecher M., Paulsson B., Jones M., Vagero M., Svensson K. (2005). A randomized, double-blind, placebo-controlled efficacy and safety study of quetiapine or lithium as monotherapy for mania in bipolar disorder. J. Clin. Psychiatry.

[42] Kushner S.F., Khan A., Lane R., Olson W.H. (2006). Topiramate monotherapy in the management of acute mania: Results of four double-blind placebo-controlled trials. Bipolar Disord..

[43] Cipriani A., Barbui C., Salanti G., Rendell J., Brown R., Stockton S., Purgato M., Spineli L.M., Goodwin G.M., Geddes J.R. (2011). Comparative efficacy and acceptability of antimanic drugs in acute mania: A multiple-treatments meta-analysis. Lancet.

[44] Smith L.A., Cornelius V., Warnock A., Tacchi M.J., Taylor D. (2007). Pharmacological interventions for acute bipolar mania: A systematic review of randomized placebo-controlled trials. Bipolar Disord..

[45] Storosum J.G., Wohlfarth T., Schene A., Elferink A., van Zwieten B.J., van den Brink W. (2007). Magnitude of effect of lithium in short-term efficacy studies of moderate to severe manic episode. Bipolar Disord..

[46] Bowden C.L., Mosolov S., Hranov L., Chen E., Habil H., Kongsakon R., Manfredi R., Lin H.N. (2010). Efficacy of valproate versus lithium in mania or mixed mania: A randomized, open 12-week trial. Int. Clin. Psychopharmacol..

[47] Bowden C., Gogus A., Grunze H., Haggstrom L., Rybakowski J., Vieta E. (2008). A 12-week, open, randomized trial comparing sodium valproate to lithium in patients with bipolar I disorder suffering from a manic episode. Int. Clin. Psychopharmacol..

[48] Berk M., Ichim L., Brook S. (1999). Olanzapine compared to lithium in mania: A double-blind randomized controlled trial. Int. Clin. Psychopharmacol..

[49] Segal J., Berk M., Brook S. (1998). Risperidone compared with both lithium and haloperidol in mania: A double-blind randomized controlled trial. Clin. Neuropharmacol..

[50] Niufan G., Tohen M., Qiuqing A., Fude Y., Pope E., McElroy H., Ming L., Gaohua W., Xinbao Z., Huichun L. (2008). Olanzapine versus lithium in the acute treatment of bipolar mania: A double-blind, randomized, controlled trial. J. Affect. Disord..

[51] Li H., Ma C., Wang G., Zhu X., Peng M., Gu N. (2008). Response and remission rates in Chinese patients with bipolar mania treated for 4 weeks with either quetiapine or lithium: A randomized and double-blind study. Curr. Med. Res. Opin..

[52] Shafti S.S., Shahveisi B. (2008). Comparison between lithium and valproate in the treatment of acute mania. J. Clin. Psychopharmacol..

[53] Shafti S.S. (2010). Olanzapine vs. lithium in management of acute mania. J. Affect. Disord..

[54] Pal Singh G. (2008). A double-blind comparative study of clinical efficacy of verapamil versus lithium in acute mania. Int. J. Psychiatry Clin. Pract..

[55] Fountoulakis K.N., Kontis D., Gonda X., Siamouli M., Yatham L.N. (2012). Treatment of mixed bipolar states. Int. J. Neuropsychopharmacol..

[56] Malhi G.S., Tanious M., Das P., Berk M. (2012). The science and practice of lithium therapy. Aust. N. Z. J. Psychiatry.

[57] Tohen M., Jacobs T.G., Feldman P.D. (2000). Onset of action of antipsychotics in the treatment of mania. Bipolar Disord..

[58] Goodwin G.M., Consensus Group of the British Association for Psychopharmacology (2009). Evidence-based guidelines for treating bipolar disorder: Revised second edition—Recommendations from the British Association for Psychopharmacology. J. Psychopharmacol..

[59] Young A.H., McElroy S.L., Bauer M., Philips N., Chang W., Olausson B., Paulsson B., Brecher M., Investigators E.I. (2010). A double-blind, placebo-controlled study of quetiapine and lithium monotherapy in adults in the acute phase of bipolar depression (EMBOLDEN I). J. Clin. Psychiatry.

[60] Kim S.J., Lee Y.J., Lee Y.J., Cho S.J. (2014). Effect of quetiapine XR on depressive symptoms and sleep quality compared with lithium in patients with bipolar depression. J. Affect. Disord..

[61] Amsterdam J.D., Wang C.H., Shwarz M., Shults J. (2009). Venlafaxine versus lithium monotherapy of rapid and non-rapid cycling patients with bipolar II major depressive episode: A randomized, parallel group, open-label trial. J. Affect. Disord..

[62] Suppes T., Marangell L.B., Bernstein I.H., Kelly D.I., Fischer E.G., Zboyan H.A., Snow D.E., Martinez M., Al Jurdi R., Shivakumar G. (2008). A single blind comparison of lithium and lamotrigine for the treatment of bipolar II depression. J. Affect. Disord..

[63] Calabrese J.R., Bowden C.L., Sachs G., Yatham L.N., Behnke K., Mehtonen O.P., Montgomery P., Ascher J., Paska W., Earl N. (2003). A placebo-controlled 18-month trial of lamotrigine and lithium maintenance treatment in recently depressed patients with bipolar I disorder. J. Clin. Psychiatry.

[64] Cipriani A., Hawton K., Stockton S., Geddes J.R. (2013). Lithium in the prevention of suicide in mood disorders: Updated systematic review and meta-analysis. BMJ.

[65] Grandjean E.M., Aubry J.M. (2009). Lithium: Updated human knowledge using an evidence-based approach: Part I: Clinical efficacy in bipolar disorder. CNS Drugs.

[66] Geddes J.R., Goodwin G.M., Rendell J., Azorin J.M., Cipriani A., Ostacher M.J., Morriss R., Alder N., Juszczak E., BALANCE Investigators and Collaborators (2010). Lithium plus valproate combination therapy versus monotherapy for relapse prevention in bipolar I disorder (BALANCE): A randomised open-label trial. Lancet.

[67] Weisler R.H., Nolen W.A., Neijber A., Hellqvist A., Paulsson B., Trial 144 Study Investigators (2011). Continuation of quetiapine versus switching to placebo or lithium for maintenance treatment of bipolar I disorder (Trial 144: A randomized controlled study). J. Clin. Psychiatry.

[68] Fornaro M., Nardi A.E., De Berardis D., Carta M.G. (2016). Experimental drugs for bipolar psychosis. Expert Opin. Investig. Drugs.

[69] Prien R.F., Caffey E.M., Klett C.J. (1972). Comparison of lithium carbonate and chlorpromazine in the treatment of mania. Report of the Veterans Administration and National Institute of Mental Health Collaborative Study Group. Arch. Gen. Psychiatry.

[70] Swann A.C., Bowden C.L., Calabrese J.R., Dilsaver S.C., Morris D.D. (2002). Pattern of response to divalproex, lithium, or placebo in four naturalistic subtypes of mania. Neuropsychopharmacology.

[71] De Sousa R.T., Busnello J.V., Forlenza O.V., Zanetti M.V., Soeiro-de-Souza M.G., van de Bilt M.T., Moreno R.A., Zarate C.A., Gattaz W.F., Machado-Vieira R. (2012). Early improvement of psychotic symptoms with lithium monotherapy as a predictor of later response in mania. J. Psychiatr. Res..

[72] Baldessarini R.J., Tondo L., Davis P., Pompili M., Goodwin F.K., Hennen J. (2006). Decreased risk of suicides and attempts during long-term lithium treatment: A meta-analytic review. Bipolar Disord..

[73] Baldessarini R.J., Tondo L. (2009). Suicidal risks during treatment of bipolar disorder patients with lithium versus anticonvulsants. Pharmacopsychiatry.

[74] Mann J.J., Waternaux C., Haas G.L., Malone K.M. (1999). Toward a clinical model of suicidal behavior in psychiatric patients. Am. J. Psychiatry.

[75] Muller-Oerlinghausen B., Lewitzka U. (2010). Lithium reduces pathological aggression and suicidality: A mini-review. Neuropsychobiology.

[76] Jimenez E., Arias B., Mitjans M., Goikolea J.M., Roda E., Saiz P.A., Garcia-Portilla M.P., Buron P., Bobes J., Oquendo M.A. (2013). Genetic variability at IMPA2, INPP1 and GSK3β increases the risk of suicidal behavior in bipolar patients. Eur. Neuropsychopharmacol..

[77] Malhi G.S., Tanious M., Das P., Coulston C.M., Berk M. (2013). Potential mechanisms of action of lithium in bipolar disorder. Current understanding. CNS Drugs.

[78] Carter C.J. (2007). Multiple genes and factors associated with bipolar disorder converge on growth factor and stress activated kinase pathways controlling translation initiation: Implications for oligodendrocyte viability. Neurochem. Int..

[79] Martinowich K., Schloesser R.J., Manji H.K. (2009). Bipolar disorder: From genes to behavior pathways. J. Clin. Investig..

[80] Serretti A., Benedetti F., Mandelli L., Calati R., Caneva B., Lorenzi C., Fontana V., Colombo C., Smeraldi E. (2008). Association between GSK-3β-50T/C polymorphism and personality and psychotic symptoms in mood disorders. Psychiatry Res..

[81] Lee Y.J., Kim Y.K. (2011). The impact of glycogen synthase kinase 3β gene on psychotic mania in bipolar disorder patients. Prog. Neuropsychopharmacol. Biol. Psychiatry.

[82] De Sousa R.T., Zanetti M.V., Talib L.L., Serpa M.H., Chaim T.M., Carvalho A.F., Brunoni A.R., Busatto G.F., Gattaz W.F., Machado-Vieira R. (2015). Lithium increases platelet serine-9 phosphorylated GSK-3β levels in drug-free bipolar disorder during depressive episodes. J. Psychiatr. Res..

[83] Iwahashi K., Nishizawa D., Narita S., Numajiri M., Murayama O., Yoshihara E., Onozawa Y., Nagahori K., Fukamauchi F., Ikeda K. (2014). Haplotype analysis of GSK-3β gene polymorphisms in bipolar disorder lithium responders and nonresponders. Clin. Neuropharmacol..

[84] Jang Y., Lee S.H., Lee B., Jung S., Khalid A., Uchida K., Tominaga M., Jeon D., Oh U. (2015). TRPM2, a susceptibility gene for bipolar disorder, regulates glycogen synthase kinase-3 activity in the brain. J. Neurosci..

[85] Dwivedi Y. (2009). Brain-derived neurotrophic factor: Role in depression and suicide. Neuropsychiatr. Dis. Treat..

[86] Cunha A.B., Frey B.N., Andreazza A.C., Goi J.D., Rosa A.R., Goncalves C.A., Santin A., Kapczinski F. (2006). Serum brain-derived neurotrophic factor is decreased in bipolar disorder during depressive and manic episodes. Neurosci. Lett..

[87] Machado-Vieira R., Dietrich M.O., Leke R., Cereser V.H., Zanatto V., Kapczinski F., Souza D.O., Portela L.V., Gentil V. (2007). Decreased plasma brain derived neurotrophic factor levels in unmedicated bipolar patients during manic episode. Biol. Psychiatry.

[88] Vincze I., Perroud N., Buresi C., Baud P., Bellivier F., Etain B., Fournier C., Karege F., Matthey M.L., Preisig M. (2008). Association between brain-derived neurotrophic factor gene and a severe form of bipolar disorder, but no interaction with the serotonin transporter gene. Bipolar Disord..

[89] Post R.M. (2007). Role of BDNF in bipolar and unipolar disorder: Clinical and theoretical implications. J. Psychiatr. Res..

[90] Emamghoreishi M., Keshavarz M., Nekooeian A.A. (2015). Acute and chronic effects of lithium on BDNF and GDNF mRNA and protein levels in rat primary neuronal, astroglial and neuroastroglia cultures. Iran. J. Basic Med. Sci..

[91] Neher E., Sakaba T. (2008). Multiple roles of calcium ions in the regulation of neurotransmitter release. Neuron.

[92] Gigante A.D., Young L.T., Yatham L.N., Andreazza A.C., Nery F.G., Grinberg L.T., Heinsen H., Lafer B. (2011). Morphometric post-mortem studies in bipolar disorder: Possible association with oxidative stress and apoptosis. Int. J. Neuropsychopharmacol..

[93] Konradi C., Eaton M., MacDonald M.L., Walsh J., Benes F.M., Heckers S. (2004). Molecular evidence for mitochondrial dysfunction in bipolar disorder. Arch. Gen. Psychiatry.

[94] Simon N.M., Smoller J.W., McNamara K.L., Maser R.S., Zalta A.K., Pollack M.H., Nierenberg A.A., Fava M., Wong K.K. (2006). Telomere shortening and mood disorders: Preliminary support for a chronic stress model of accelerated aging. Biol. Psychiatry.

[95] Macedo D.S., de Lucena D.F., Queiroz A.I., Cordeiro R.C., Araujo M.M., Sousa F.C., Vasconcelos S.M., Hyphantis T.N., Quevedo J., McIntyre R.S. (2013). Effects of lithium on oxidative stress and behavioral alterations induced by lisdexamfetamine dimesylate: Relevance as an animal model of mania. Prog. Neuropsychopharmacol. Biol. Psychiatry.

[96] Nciri R., Desmoulin F., Allagui M.S., Murat J.C., Feki A.E., Vincent C., Croute F. (2013). Neuroprotective effects of chronic exposure of SH-SY5Y to low lithium concentration involve glycolysis stimulation, extracellular pyruvate accumulation and resistance to oxidative stress. Int. J. Neuropsychopharmacol..

[97] Wang H.M., Zhang T., Li Q., Huang J.K., Chen R.F., Sun X.J. (2013). Inhibition of glycogen synthase kinase-3β by lithium chloride suppresses 6-hydroxydopamine-induced inflammatory response in primary cultured astrocytes. Neurochem. Int..

[98] Watanabe S., Iga J., Nishi A., Numata S., Kinoshita M., Kikuchi K., Nakataki M., Ohmori T. (2014). Microarray analysis of global gene expression in leukocytes following lithium treatment. Hum. Psychopharmacol..

[99] Myint A.M., Kim Y.K. (2014). Network beyond IDO in psychiatric disorders: Revisiting neurodegeneration hypothesis. Prog. Neuropsychopharmacol. Biol. Psychiatry.

[100] Kim Y.K., Jung H.G., Myint A.M., Kim H., Park S.H. (2007). Imbalance between pro-inflammatory and anti-inflammatory cytokines in bipolar disorder. J. Affect. Disord..

[101] Rizak J., Tan H., Zhu H., Wang J.F. (2014). Chronic treatment with the mood-stabilizing drug lithium up-regulates nuclear factor E2-related factor 2 in rat pheochromocytoma PC12 cells in vitro. Neuroscience.

[102] De Sousa R.T., Zarate C.A., Zanetti M.V., Costa A.C., Talib L.L., Gattaz W.F., Machado-Vieira R. (2014). Oxidative stress in early stage Bipolar Disorder and the association with response to lithium. J. Psychiatr. Res..

[103] De Sousa R.T., Streck E.L., Zanetti M.V., Ferreira G.K., Diniz B.S., Brunoni A.R., Busatto G.F., Gattaz W.F., Machado-Vieira R. (2015). Lithium increases leukocyte mitochondrial complex I activity in bipolar disorder during depressive episodes. Psychopharmacology.

[104] Tanno M., Kuno A., Ishikawa S., Miki T., Kouzu H., Yano T., Murase H., Tobisawa T., Ogasawara M., Horio Y. (2014). Translocation of glycogen synthase kinase-3β (GSK-3β), a trigger of permeability transition, is kinase activity-dependent and mediated by interaction with voltage-dependent anion channel 2 (VDAC2). J. Biol. Chem..

[105] Ngok-Ngam P., Watcharasit P., Thiantanawat A., Satayavivad J. (2013). Pharmacological inhibition of GSK3 attenuates DNA damage-induced apoptosis via reduction of p53 mitochondrial translocation and Bax oligomerization in neuroblastoma SH-SY5Y cells. Cell. Mol. Biol. Lett..

[106] Keshavarz M., Emamghoreishi M., Nekooeian A.A., Warsh J.J., Zare H.R. (2013). Increased bcl-2 protein levels in rat primary astrocyte culture following chronic lithium treatment. Iran. J. Med. Sci..

[107] Rajkowska G., Clarke G., Mahajan G., Licht C.M., van de Werd H.J., Yuan P., Stockmeier C.A., Manji H.K., Uylings H.B. (2016). Differential effect of lithium on cell number in the hippocampus and prefrontal cortex in adult mice: A stereological study. Bipolar Disord..

[108] Meffre D., Massaad C., Grenier J. (2015). Lithium chloride stimulates PLP and MBP expression in oligodendrocytes via Wnt/β-catenin and Akt/CREB pathways. Neuroscience.

[109] Malhi G.S., Outhred T. (2016). Therapeutic mechanisms of lithium in Bipolar Disorder: Recent advances and current understanding. CNS Drugs.

[110] Sarkar S., Floto R.A., Berger Z., Imarisio S., Cordenier A., Pasco M., Cook L.J., Rubinsztein D.C. (2005). Lithium induces autophagy by inhibiting inositol monophosphatase. J. Cell Biol..

[111] Tsui M.M., Tai W.C., Wong W.Y., Hsiao W.L. (2012). Selective G2/M arrest in a p53(Val135)-transformed cell line induced by lithium is mediated through an intricate network of MAPK and β-catenin signaling pathways. Life Sci..

[112] Zaeri S., Farjadian S., Emamghoreishi M. (2015). Decreased levels of canonical transient receptor potential channel 3 protein in the rat cerebral cortex after chronic treatment with lithium or valproate. Res. Pharm. Sci..

[113] Heinrich A., von der Heyde A.S., Boer U., Phu do T., Tzvetkov M., Oetjen E. (2013). Lithium enhances CRTC oligomer formation and the interaction between the CREB coactivators CRTC and CBP—Implications for CREB-dependent gene transcription. Cell Signal..

[114] Yuksel C., Ongur D. (2010). Magnetic resonance spectroscopy studies of glutamate-related abnormalities in mood disorders. Biol. Psychiatry.

[115] Chitty K.M., Lagopoulos J., Lee R.S., Hickie I.B., Hermens D.F. (2013). A systematic review and meta-analysis of proton magnetic resonance spectroscopy and mismatch negativity in bipolar disorder. Eur. Neuropsychopharmacol..

[116] McCullumsmith R.E., Kristiansen L.V., Beneyto M., Scarr E., Dean B., Meador-Woodruff J.H. (2007). Decreased NR1, NR2A, and SAP102 transcript expression in the hippocampus in bipolar disorder. Brain Res..

[117] Berk M., Dodd S., Kauer-Sant’anna M., Malhi G.S., Bourin M., Kapczinski F., Norman T. (2007). Dopamine dysregulation syndrome: Implications for a dopamine hypothesis of bipolar disorder. Acta Psychiatr. Scand. Suppl..

[118] Daniele S., Da Pozzo E., Abelli M., Panighini A., Pini S., Gesi C., Lari L., Cardini A., Cassano G.B., Martini C. (2012). Platelet uptake of GABA and glutamate in patients with bipolar disorder. Bipolar Disord..

[119] Gos T., Steiner J., Bielau H., Dobrowolny H., Gunther K., Mawrin C., Krzyzanowski M., Hauser R., Brisch R., Bernstein H.G. (2012). Differences between unipolar and bipolar I depression in the quantitative analysis of glutamic acid decarboxylase-immunoreactive neuropil. Eur. Arch. Psychiatry Clin. Neurosci..

[120] Brady R.O., McCarthy J.M., Prescot A.P., Jensen J.E., Cooper A.J., Cohen B.M., Renshaw P.F., Ongur D. (2013). Brain gamma-aminobutyric acid (GABA) abnormalities in bipolar disorder. Bipolar Disord..

[121] Wakita M., Nagami H., Takase Y., Nakanishi R., Kotani N., Akaike N. (2015). Modifications of excitatory and inhibitory transmission in rat hippocampal pyramidal neurons by acute lithium treatment. Brain Res. Bull..

[122] Higgins G.A., Allyn-Feuer A., Barbour E., Athey B.D. (2015). A glutamatergic network mediates lithium response in bipolar disorder as defined by epigenome pathway analysis. Pharmacogenomics.

[123] Mavrikaki M., Schintu N., Kastellakis A., Nomikos G.G., Svenningsson P., Panagis G. (2014). Effects of lithium and aripiprazole on brain stimulation reward and neuroplasticity markers in the limbic forebrain. Eur. Neuropsychopharmacol..

[124] Calabrese B., Halpain S. (2014). Lithium prevents aberrant NMDA-induced F-actin reorganization in neurons. Neuroreport.

[125] Zanetti M.V., Otaduy M.C., de Sousa R.T., Gattaz W.F., Busatto G.F., Leite C.C., Machado-Vieira R. (2014). Bimodal effect of lithium plasma levels on hippocampal glutamate concentrations in bipolar II depression: A pilot study. Int. J. Neuropsychopharmacol..

[126] Ago Y., Tanaka T., Kita Y., Tokumoto H., Takuma K., Matsuda T. (2012). Lithium attenuates methamphetamine-induced hyperlocomotion and behavioral sensitization via modulation of prefrontal monoamine release. Neuropharmacology.

[127] Van Enkhuizen J., Milienne-Petiot M., Geyer M.A., Young J.W. (2015). Modeling bipolar disorder in mice by increasing acetylcholine or dopamine: Chronic lithium treats most, but not all features. Psychopharmacology.

[128] Jimenez E., Nunez E., Ibanez I., Zafra F., Aragon C., Gimenez C. (2015). Glycine transporters GlyT1 and GlyT2 are differentially modulated by glycogen synthase kinase 3β. Neuropharmacology.

[129] Rock P., Goodwin G., Harmer C., Wulff K. (2014). Daily rest-activity patterns in the bipolar phenotype: A controlled actigraphy study. Chronobiol. Int..

[130] Milhiet V., Etain B., Boudebesse C., Bellivier F. (2011). Circadian biomarkers, circadian genes and bipolar disorders. J. Physiol. Paris.

[131] Robillard R., Naismith S.L., Rogers N.L., Scott E.M., Ip T.K., Hermens D.F., Hickie I.B. (2013). Sleep-wake cycle and melatonin rhythms in adolescents and young adults with mood disorders: Comparison of unipolar and bipolar phenotypes. Eur. Psychiatry.

[132] McCarthy M.J., Wei H., Marnoy Z., Darvish R.M., McPhie D.L., Cohen B.M., Welsh D.K. (2013). Genetic and clinical factors predict lithium’s effects on PER2 gene expression rhythms in cells from bipolar disorder patients. Transl. Psychiatry.

[133] McCarthy M.J., Nievergelt C.M., Kelsoe J.R., Welsh D.K. (2012). A survey of genomic studies supports association of circadian clock genes with bipolar disorder spectrum illnesses and lithium response. PLoS ONE.

[134] Schnell A., Sandrelli F., Ranc V., Ripperger J.A., Brai E., Alberi L., Rainer G., Albrecht U. (2015). Mice lacking circadian clock components display different mood-related behaviors and do not respond uniformly to chronic lithium treatment. Chronobiol. Int..

[135] Taylor V., MacQueen G. (2006). Associations between bipolar disorder and metabolic syndrome: A review. J. Clin. Psychiatry.

[136] Tsigos C., Chrousos G.P. (2002). Hypothalamic-pituitary-adrenal axis, neuroendocrine factors and stress. J. Psychosom. Res..

[137] Kovzun E.I., Lukashenya O.S., Pushkarev V.M., Mikosha A.S., Tron’ko N.D. (2014). Effect of ions of potassium and lithium on NO synthase expression in the human adrenal cortex. Bull. Exp. Biol. Med..

[138] Kim Y.K., Na K.S., Hwang J.A., Yoon H.K., Lee H.J., Hahn S.W., Lee B.H., Jung H.Y. (2013). High insulin-like growth factor-1 in patients with bipolar I disorder: A trait marker?. J. Affect. Disord..

[139] Lee B.H., Kim Y.K. (2012). Increased plasma VEGF levels in major depressive or manic episodes in patients with mood disorders. J. Affect. Disord..

[140] Strakowski S.M., DelBello M.P., Zimmerman M.E., Getz G.E., Mills N.P., Ret J., Shear P., Adler C.M. (2002). Ventricular and periventricular structural volumes in first-versus multiple-episode bipolar disorder. Am. J. Psychiatry.

[141] Brambilla P., Harenski K., Nicoletti M., Mallinger A.G., Frank E., Kupfer D.J., Keshavan M.S., Soares J.C. (2001). MRI study of posterior fossa structures and brain ventricles in bipolar patients. J. Psychiatr. Res..

[142] Lim C.S., Baldessarini R.J., Vieta E., Yucel M., Bora E., Sim K. (2013). Longitudinal neuroimaging and neuropsychological changes in bipolar disorder patients: Review of the evidence. Neurosci. Biobehav. Rev..

[143] Lyoo I.K., Sung Y.H., Dager S.R., Friedman S.D., Lee J.Y., Kim S.J., Kim N., Dunner D.L., Renshaw P.F. (2006). Regional cerebral cortical thinning in bipolar disorder. Bipolar Disord..

[144] Beyer J.L., Krishnan K.R. (2002). Volumetric brain imaging findings in mood disorders. Bipolar Disord..

[145] Blumberg H.P., Fredericks C., Wang F., Kalmar J.H., Spencer L., Papademetris X., Pittman B., Martin A., Peterson B.S., Fulbright R.K. (2005). Preliminary evidence for persistent abnormalities in amygdala volumes in adolescents and young adults with bipolar disorder. Bipolar Disord..

[146] Nortje G., Stein D.J., Radua J., Mataix-Cols D., Horn N. (2013). Systematic review and voxel-based meta-analysis of diffusion tensor imaging studies in bipolar disorder. J. Affect. Disord..

[147] Vederine F.E., Wessa M., Leboyer M., Houenou J. (2011). A meta-analysis of whole-brain diffusion tensor imaging studies in bipolar disorder. Prog. Neuropsychopharmacol. Biol. Psychiatry.

[148] Chen X., Wen W., Malhi G.S., Ivanovski B., Sachdev P.S. (2007). Regional gray matter changes in bipolar disorder: A voxel-based morphometric study. Aust. N. Z. J. Psychiatry.

[149] Wijeratne C., Sachdev S., Wen W., Piguet O., Lipnicki D.M., Malhi G.S., Mitchell P.B., Sachdev P.S. (2013). Hippocampal and amygdala volumes in an older bipolar disorder sample. Int. Psychogeriatr..

[150] Eker C., Simsek F., Yilmazer E.E., Kitis O., Cinar C., Eker O.D., Coburn K., Gonul A.S. (2014). Brain regions associated with risk and resistance for bipolar I disorder: A voxel-based MRI study of patients with bipolar disorder and their healthy siblings. Bipolar Disord..

[151] Bearden C.E., Thompson P.M., Dalwani M., Hayashi K.M., Lee A.D., Nicoletti M., Trakhtenbroit M., Glahn D.C., Brambilla P., Sassi R.B. (2007). Greater cortical gray matter density in lithium-treated patients with bipolar disorder. Biol. Psychiatry.

[152] Van der Schot A.C., Vonk R., Brans R.G., van Haren N.E., Koolschijn P.C., Nuboer V., Schnack H.G., van Baal G.C., Boomsma D.I., Nolen W.A. (2009). Influence of genes and environment on brain volumes in twin pairs concordant and discordant for bipolar disorder. Arch. Gen. Psychiatry.

[153] Takahashi T., Malhi G.S., Wood S.J., Yucel M., Walterfang M., Kawasaki Y., Suzuki M., Pantelis C. (2010). Gray matter reduction of the superior temporal gyrus in patients with established bipolar I disorder. J. Affect. Disord..

[154] Germana C., Kempton M.J., Sarnicola A., Christodoulou T., Haldane M., Hadjulis M., Girardi P., Tatarelli R., Frangou S. (2010). The effects of lithium and anticonvulsants on brain structure in bipolar disorder. Acta Psychiatr. Scand..

[155] Radenbach K., Flaig V., Schneider-Axmann T., Usher J., Reith W., Falkai P., Gruber O., Scherk H. (2010). Thalamic volumes in patients with bipolar disorder. Eur. Arch. Psychiatry Clin. Neurosci..

[156] Hajek T., Cullis J., Novak T., Kopecek M., Hoschl C., Blagdon R., O’Donovan C., Bauer M., Young L.T., Macqueen G. (2012). Hippocampal volumes in bipolar disorders: Opposing effects of illness burden and lithium treatment. Bipolar Disord..

[157] Hajek T., Bauer M., Simhandl C., Rybakowski J., O’Donovan C., Pfennig A., Konig B., Suwalska A., Yucel K., Uher R. (2014). Neuroprotective effect of lithium on hippocampal volumes in bipolar disorder independent of long-term treatment response. Psychol. Med..

[158] Benedetti F., Bollettini I., Barberi I., Radaelli D., Poletti S., Locatelli C., Pirovano A., Lorenzi C., Falini A., Colombo C. (2013). Lithium and GSK3-β promoter gene variants influence white matter microstructure in bipolar disorder. Neuropsychopharmacology.

[159] Benedetti F., Poletti S., Radaelli D., Locatelli C., Pirovano A., Lorenzi C., Vai B., Bollettini I., Falini A., Smeraldi E. (2015). Lithium and GSK-3β promoter gene variants influence cortical gray matter volumes in bipolar disorder. Psychopharmacology.

[160] Sun Y.R., Herrmann N., Scott C.J.M., Black S.E., Khan M.M., Lanctot K.L. (2018). Global grey matter volume in adult bipolar patients with and without lithium treatment: A meta-analysis. J. Affect. Disord..

[161] Tighe S.K., Mahon P.B., Potash J.B. (2011). Predictors of lithium response in bipolar disorder. Ther. Adv. Chronic Dis..

